# Acute lower limb ischemia in an ICU admitted patient diagnosed with the COVID‐19: A case report

**DOI:** 10.1002/ccr3.5146

**Published:** 2021-12-04

**Authors:** Armin Sadeghi, Mohammadreza Moselmi

**Affiliations:** ^1^ Tuberculosis and Lung Disease Research Center Tabriz University of Medical Sciences Tabriz Iran; ^2^ Department of Internal Medicine School of Medicine Tabriz University of Medical Sciences Tabriz Iran

**Keywords:** arterial thrombosis, COVID‐19, limb ischemia

## Abstract

Thromboembolic events have been reported in the hospitalized patient since the beginning of the COVID‐19 pandemics. ICU‐admitted patients demonstrated a significantly higher risk of developing VTE. Although evidence of arterial thrombosis was less common in ICU‐admitted patients, consequences were typically more severe, including limb loss and death. This study reports another ICU‐admitted patient with lower extremity arterial thrombosis diagnosed with COVID‐19.

## Graphical abstract

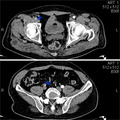

## 1 INTRODUCTION

The coronavirus disease 2019, caused by severe acute respiratory syndrome coronavirus 2 (SARS‐CoV‐2), is a rapidly evolving health crisis. COVID‐19 is also manifested with hypercoagulability, pulmonary intravascular coagulation, microangiopathy, venous thromboembolism (VTE), and arterial thrombosis.^1^ Since the beginning of the COVID‐19 pandemics, many cases of thromboembolic events have been reported.^1^, ^2^, ^3^, ^4^ The prevalence of thrombosis among patients with COVID‐19 is not fully established. Studies showed that most of them are venous thromboembolism.^4^, ^5^ Arterial events have commonly been reported in the forms of acute coronary syndrome.^5^, ^6^ Patients in the ICU demonstrated a significantly higher risk of developing VTE, even when receiving pharmacologic thromboprophylaxis. Although evidence of arterial thrombosis was less common, its consequences were typically more severe, including limb loss and death even in young individuals.^4^ This study reports another ICU (intensive care unit) admitted COVID‐19 patient with Iliac and lower limb arterial thrombosis.

## 2 CASE PRESENTATION

A 40‐year‐old male patient without any past medical history was admitted to the COVID‐19 ICU with the complaint of fever, myalgia, and progressive dyspnea over four days. Vital signs on the day of admission revealed blood pressure of 110/60 mmHg, heart rate of 87, respiratory rate of 25, low‐grade fever, and oxygen saturation of 85% on room air. Chest computerized tomography scan (CT scan) showed peripheral bilateral patchy ground‐glass infiltrations, which is suggestive for COVID‐19 pneumonia and venous blood gas information (ph:7/41 PO_2_:54 mm Hg PCO_2_: 35 mmHg HCO_3_:20 mmol/dl) and O_2_ saturation of 85% revealed the patient acute respiratory distress syndrome (ARDS). Initial laboratory evaluations showed as follows: hemoglobin 14 g/dl, total leukocyte count 9,800 per microliter, absolute neutrophil count 7,100 per microliter absolute lymphocyte count 2,100 per microliter, platelets 330,000 per microliter, BUN 28 mg/dl, serum creatinine 0.9 mg/dl, prothrombin time 12 s, international normalized ratio 1 s, partial thromboplastin time 38s, lactate dehydrogenase 1,200 μ/L, and C‐reactive protein was 86 mg/dl. An electrocardiogram showed sinus tachycardia of 106 beats per minute. The patient was started on remdesivir, dexamethasone, and heparin for prophylaxis.

**FIGURE 1:**
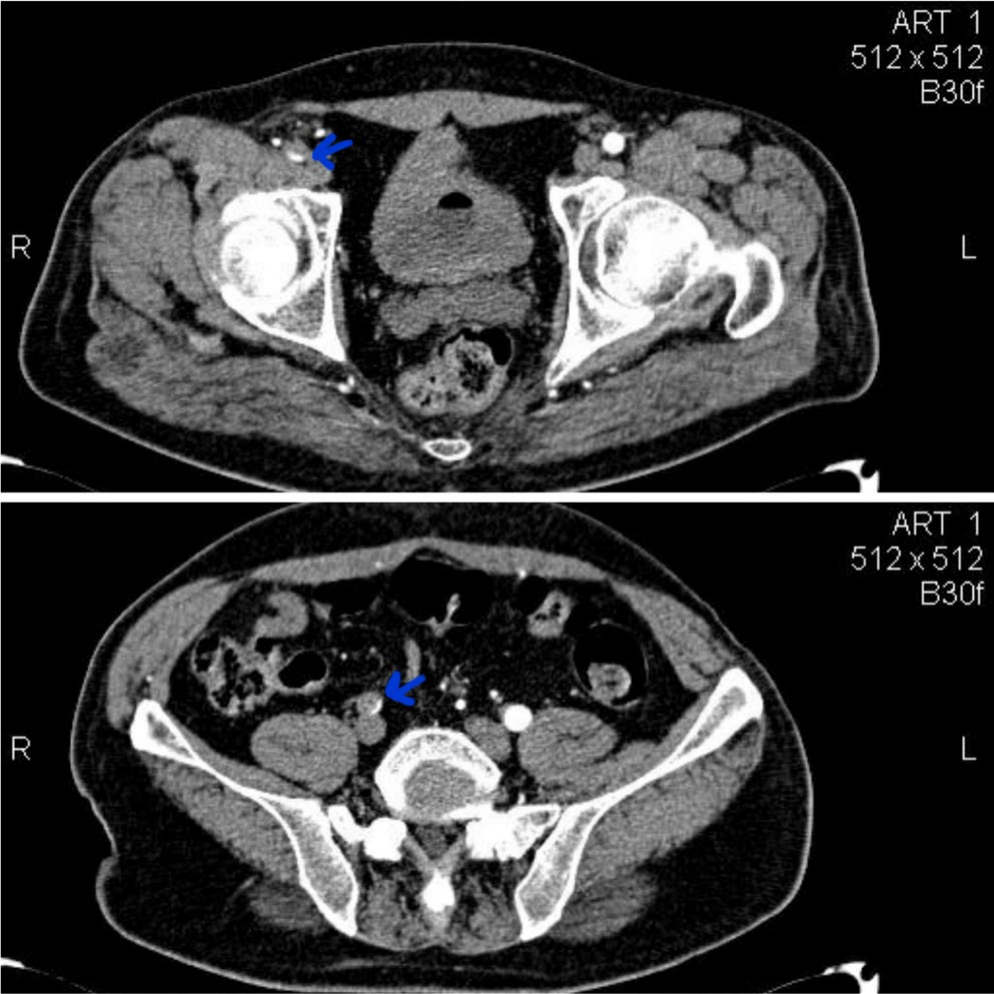
CT angiography showed thrombotic occlusion at the right common iliac artery, right external iliac artery, and common femoral artery (blue arrows)

**FIGURE 2:**
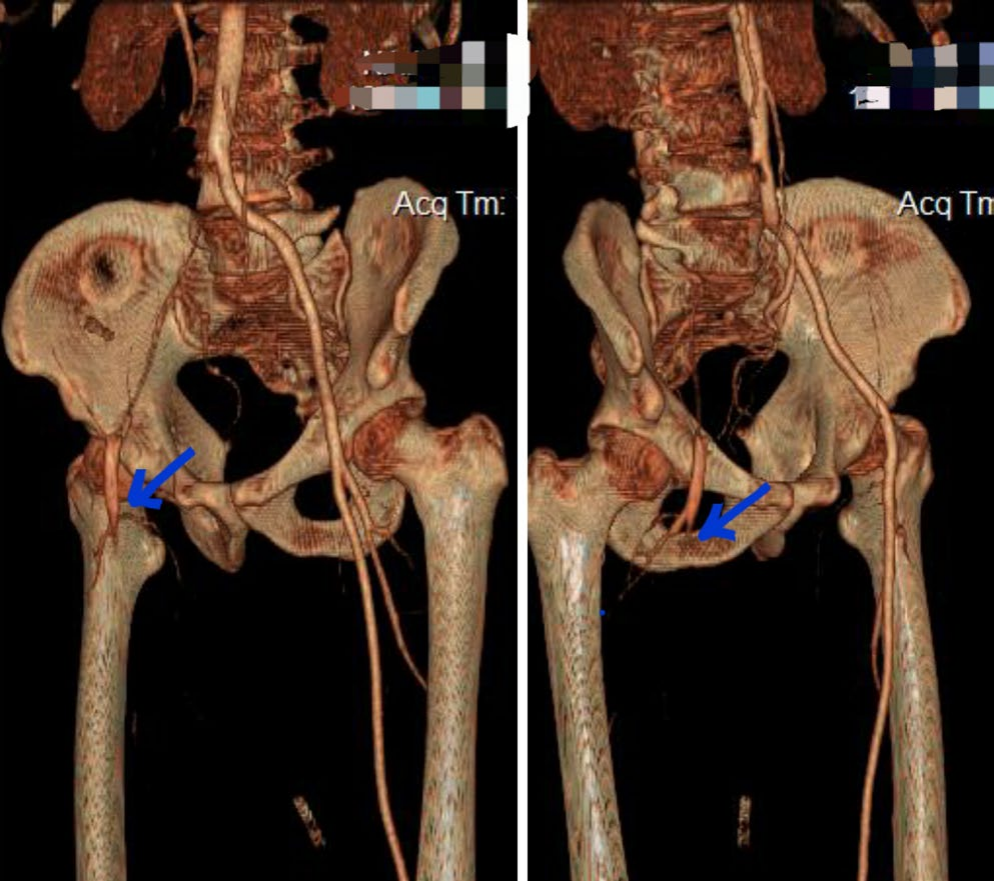
Reconstructed 3D CT angiography shows a cutoff distal to the common iliac artery (blue arrows)

On the second day of hospitalization, the patient complained about a sudden severe pain in his right leg. On examination, he had profound weakness in the right leg, mild weakness in the left leg, and absent pulses. The findings of laboratory data (complete blood counts and PT, PTT, INR, and liver and kidney function tests) were unremarkable. Doppler ultrasonography reported intraluminal thrombus in the distal part of the right common iliac artery. CT angiography showed thrombotic occlusion at the right common iliac artery, right external iliac artery, and common femoral artery (Figures 1, 2).

Lupus anticoagulant antibody, antiphospholipid antibody, anticardiolipin antibodies, anti beta2 glycoprotein, anti‐nuclear antibody (ANA), P‐ANCA, C‐ANCA, and c3, c4, CH50 complement level were in the normal range.

The patient was started on therapeutic anticoagulation with unfractionated heparin. Angiography of bilateral iliac and lower extremity arteries showed thrombotic occlusion of right common iliac artery, right external iliac artery, right superficial femoral artery, right popliteal artery, and partial thrombolysis at left common iliac artery.

Revascularization and thrombolysis by injecting tissue plasminogen activator were not sufficient, and the patient underwent leg amputation one day after angiography.

## 3 DISCUSSION

Since September 2019, during the COVID‐19 pandemics, many cases of thromboembolic events have been reported.^1^, ^2^, ^3^, ^4^ The prevalence of thrombosis among patients with COVID‐19 is not fully established, but studies showed that most of them are venous thromboembolism.^5^ A study revealed that the cumulative incidence of a composite outcome of vascular events (acute pulmonary embolism, deep‐vein thrombosis, ischemic stroke, myocardial infarction, and systemic arterial embolism) was 31%, the incidence of venous thromboembolism was 27%, and the incidence of arterial events was 3.7%, among patients in the ICU.^7^ Another systematic review shows that patients in the ICU demonstrated a significantly higher risk of developing VTE, even when receiving pharmacologic thromboprophylaxis and arterial thrombosis was less common in these patients.^4^ Arterial thrombosis has also been reported within coronary arteries and within the brain in COVID‐19 patients.^5^, ^8^, ^9^, ^10^ Studies showed that venous thromboembolism has also been a significant cause of morbidity and mortality in patients with COVID‐19, both in the general inpatient and in the intensive care unit (ICU) setting, and even in patients receiving therapeutic anticoagulation.^11^, ^12^, ^13^ In this study, we reported another ICU admitted COVID‐19 patient who developed arterial lower extremity thrombosis leading to acute ischemia and limb amputation, which is attributed to recent reports.

Although the pathophysiological pathway by which COVID‐19 produces thrombosis is not completely clear, some hypotheses have been elucidated to explain the correlation of COVID‐19 disease and thromboembolic events. Some authors think ample time in the ICU and prolonged mechanical ventilation may lead to a prothrombotic state,^14^ which could justify the increase in morbidity and mortality of thromboembolism in patients with COVID‐19 in the intensive care unit (ICU).

In severe COVID‐19, hypercoagulability and endothelial activation and complement activation because of infection of endothelial cells cause endothelial damage, coagulopathy, and complement‐induced thrombosis.^15^, ^16^, ^17^ The direct procoagulant activity of SARS‐CoV‐2 is through main proteinase (Mpro)—also known as 3Clpro—catalyzes the viral polyprotein processing, which is a necessary procedure for SARS‐CoV‐2 infection.^18^ Mpro is unique in the virus and not found in the host cells. Mpro from SARS‐CoV‐2 shares structural similarities with the active site of FXa and thrombin, and it may activate blood coagulation.^19^ Hypercoagulability in patients with COVID‐19 is also can be initiated by activation of the contact system, which is composed of three groups of serine proteinases: (1) plasma prekallikrein (PPK), (2) the clotting factors XII (FXII) and XI (FXI), and (3) the nonenzymatic cofactor "high‐molecular‐weight kininogen" (HMWK).^20^ Cleavage of free HMWK by plasma kallikrein (PK) releases bradykinin, a potent inflammatory mediator and an activator of the complement and contact system. Contact system activation also leads to FXII activation (FXIIa) and ignition of coagulation and thrombosis cascade.^21^, ^22^

In our study, assessment about vasculitis, antiphospholipid antibody syndrome, systemic lupus erythematosus, and other common causes of arterial thrombosis was unremarkable. So probably, COVID‐19 can be considered as the probable cause of thromboembolism in our patient through these mechanisms.

This study reported a COVID‐19 ICU‐admitted patient who developed arterial lower extremity thrombosis leading to acute ischemia and limb amputation. Our case and several reports of thromboembolic events, especially lower extremity arterial thrombosis, which is characterized by a greater clot burden and a more dire prognosis among COVID‐19 hospitalized patients, reveal that healthcare providers should be aware of life‐threatening thromboembolic events associated with COVID‐19 so that prompt and appropriate intervention can be undertaken.

## CONFLICT OF INTEREST

None.

## AUTHOR CONTRIBUTIONS

Armin Sadeghi the conception and design of the study, and final approval of the version to be submitted. Mohammadreza Moselmi the conception and design of the study, drafting the article, and final approval of the version to be submitted.

## ETHICAL APPROVAL

This study was performed according to the principles outlined by the World Medical Association's Declaration of Helsinki on experimentation involving human subjects, as revised in 2000 and has been approved by the ethics committee of the Tabriz University of Medical Sciences.

## CONSENT

Written informed consent was obtained from the patient for the publication of this report and clinical images. Consent has been signed and collected in accordance with the journal's patient consent policy.

## Data Availability

The data support the findings of this study are available from the corresponding author upon reasonable request.
